# Development of diagnostic model of lung cancer based on multiple tumor markers and data mining

**DOI:** 10.18632/oncotarget.21935

**Published:** 2017-10-19

**Authors:** Zhaoxian Wang, Feifei Feng, Xiaoshan Zhou, Liju Duan, Jing Wang, Yongjun Wu, Na Wang

**Affiliations:** ^1^ College of Public Health, Zhengzhou University, Henan, China; ^2^ Division of Clinical Microbiology, Department of Laboratory Medicine, Karolinska Institute, Karolinska University Hospital, Huddinge, Sweden; ^3^ The First Affiliated Hospital of Zhengzhou University, Henan, China

**Keywords:** lung cancer, decision tree, ANN, diagnostic model, tumor marker

## Abstract

**Objective:**

To develop early intelligent discriminative model of lung cancer and evaluate the efficiency of diagnosis value.

**Methods:**

Based on the genetic polymorphism profile of CYP1A1-rs1048943, GSTM1, mEH-rs1051740, XRCC1-rs1799782 and XRCC1-rs25489 and the methylations of p16 and RASSF1A gene, and the length of telomere in the peripheral blood from 200 lung cancer patients and 200 health persons, the discriminative model was established through decision tree and ANN technique.

**Results:**

ACU of the discriminative model based on multiple tumour markers increased by about 10%; The accuracy rate of decision tree model and ANN model for testing set were 93.00% and 89.62% respectively. The ROC analysis showed the decision tree model’s AUC is 0.929 (0.894∼0.964), the ANN model’s AUC is 0.894 (0.853∼0.935). However, the classify accuracy rate and AUC of Fisher discriminatory analysis model are all about 0.7.

**Conclusion:**

The early intelligent discriminative model of lung cancer based on multiple tumor markers and data mining techniques has a higher accuracy rate and might be useful for early diagnosis of lung cancer.

## INTRODUCTION

According to WHO data, cancer is the second cause of death which has caused about one-sixth of the death (8.8 million) in 2015 worldwide. Lung cancer is the leading cause of cancer death, which led to 1.69 million people died, accounting for about 19% [1]. In China, there are approximately 600,000 people died because of lung cancer. The morbidity and mortality of lung cancer is the highest in the malignant tumors [2]. The 5-year survival rate of IA stages lung cancer was 70%, but the total rate was only about 15%, and the standardized mortality rates are expected to continue rising [3]. Therefore, improvement of the early diagnosis has great clinical significance for the prevention and treatment of lung cancer.

With the development of gene expression profiling technology and data mining technology, people could obtain and analyze the early molecular events of lung cancer, and thus expected to achieve the secondary prevention of lung cancer [4]. To date, low-dose computed tomography (CT), Auto Fluorescence Bronchoscope and Liquid-Based Cytology versus Conventional Cytology are used for the diagnosis of lung cancer, which made some progress, but still have some limitations in sensitivity, specificity and applicability. Thus, starting from the serum markers, finding susceptible and effective biomarkers have become a hot research topic. At present, many single nucleotide polymorphisms (SNPs) associated with lung cancer have been found by GWAS, Taqman probe (Taqman real time PCR) assays, DNA sequencing technology, such as CYFRA21-1 [5, 6], NSE [7], CA19-9 [8], KDM4A [9, 10], TP53 [11], KRT81 [12], etc. In epigenetic field, methylation, histone modification, RNA correlation silence, telomere are also relates with the development of lung cancer [13–16]. Multiple tumor markers are usually used to improve the detection effect of early lung cancer, because the single tumor marker isn’t reliable.

In this study, we screened the biomarkers related to genetic susceptibility and epigenic modification of relevant genes in lung cancer, and analyzed the relationship between these biomarkers and the occurrence of lung cancer and established the early intelligent diagnostic model of lung cancer was also established based on multiple tumor markers and data mining techniques. We also performed comparison between data mining and Fisher discriminatory analysis in the classification effect, explored the application value of tumor markers in the early warning of lung cancer, in order to construct the early intelligentized model for diagnosis.

## RESULTS

### General data of research objects was compared

The age difference between the case group and the control group was statistically significant (*P*<0.05), the gender difference was not statistically significant (*P*>0.05). The smoking rate of lung cancer group was higher than control group, the difference was statistically significant (*P*<0.05), seen in Table 1.

**Table 1:** General characteristics of the case and control group

	Case group(n=200)	Control group(n=200)	*χ*^*2*^*/t*	*P*
Age (±s)	59.56±10.56	53.70±13.34	4.872	<0.001
Male (n)	143	151	0.821	0.428
Smokin (n)	107	79	7.879	0.007

### Correlation between the genetic polymorphism of CYP1A1, GSTM1, GSTT1, mEH, XRCC1 and lung cancer

CYP1A1-rs1048943 GG+AG genotype, GSTM1 deletion genotype, mEH-rs1051740 mutant genotype, XRCC1-rs1799782 TT+CT genotype, XRCC1- rs25489 GG genotype showed significant correlations with lung cancer risk increased. Mutant genotype and wildtype genotype of CYP1A1- rs35463883, mEH-rs55784606, XRCC1- rs25487 gene and GSTT1 deletion genotype were no significantly difference between the case group and the control group(*P*>0.05), seen in Table 2.

**Table 2:** CYP1A1, GSTM1, GSTT1, mEH, XRCC1 gene polymorphisms and lung cancer susceptibility association analysis

Gene polymorphisms	Case group		Control group		*OR*(95%*CI*)	*OR*_*adj*_ (95 %*CI*)^*#*^
	n	%	n	%		
CYP1A1						
rs35463883						
Wildtype (TT)	59	29.5	68	34.0	1.00	1.00
Variant (CC+CT)	141	70.5	132	66.0	1.231 (0.846-1.791)	1.133 (0.773-1.661)
rs1048943						
Wildtype (AA)	90	45.0	116	57.8	1.00	1.00
Variant (GG+AG)	110	55.0	84	42.2	1.688 (1.136-2.507)^*^	1.727 (1.203-2.477)
GSTM1						
+	82	41.4	112	55.9	1.00	1.00
-	118	58.6	88	44.1	1.831 (1.232-2.723)	1.727 (1.211-2.463)
GSTT1						
+	114	56.6	122	60.9	1.00	1.00
-	86	43.4	78	39.1	1.180 (0.792-1.758)	1.284 (0.893-1.847)
mEH						
rs1051740						
Wildtype (TT)	51	25.5	76	37.9	1.00	1.00
Variant (CC+TC)	149	74.5	124	62.1	1.791 (1.168-2.745)	1.758 (1.194-2.589)^*^
rs55784606						
Wildtype (CC)	154	76.9	162	80.8	1.00	1.00
Variant (TT+CT)	46	23.1	38	19.1	1.273 (0.786-2.064)	1.436 (0.924-2.231)
XRCC1						
rs1799782						
Wildtype (CC)	86	43.0	108	53.9	1.00	1.00
Variant (TT+CT)	114	57.0	92	46.1	1.556 (1.049-2.309)	1.542 (1.083-2.196)
rs25489						
Variant (AA+GA)	180	90.4	192	95.7	1.00	1.00
Wildtype (GG)	20	9.6	8	4.3	2.667 (1.146-6.206)	2.941 (1.427-6.060)
rs25487						
Wildtype (GG)	100	50.2	107	53.5	1.00	1.00
Variant (AA+GA)	100	49.8	93	46.5	1.151 (0.777-1.704)	1.163 (0.805-1.680)

^#^: Adjusted by gender, age, smoking status; ^*^:P<0.05.

### Correlation between the methylation of p16 gene and RASSF1A gene and lung cancer

The lung cancer patient group and the control group were divided into four layers according to the quartile of two genes methylation level, the results showed that the increase of p16 gene and RASSF1A gene correlated with increasing risk of lung cancer(*P*_*trend*_<0.05); The median of two genes methylation level was divided into two layers according to the median, the results showed the level of methylation higher than the median will cause increasing risk of lung cancer as seen in Table 3.

**Table 3:** The level of p16, RASSF1A gene methylation and the risk of lung cancer

The level of gene methylation (%)		Lung cancer group	Control group	OR (95%CI)^*^	P^*^
P16 is classified by quartile	First quartile	35	65	1	—
	Second quartile	52	48	1.856 (1.018∼3.382)	0.043
	Third quartile	57	44	2.310 (1.270∼4.202)	0.006
	Fourth quartile	56	43	2.079 (1.140∼3.791)	0.017
P^**^		0.006		**—**	**—**
P trend		**—**	**—**	0.002	**—**
P16 is classified by median	≤Median	87	113	1	**—**
	>Median	113	87	1.597 (1.052∼2.422)	0.028
P^**^		0.009		**—**	**—**
RASSF1A is classified by quartile	First quartile	38	62	1	**—**
	Second quartile	50	49	1.492 (0.822∼2.708)	0.189
	Third quartile	58	43	1.976 (1.088∼3.591)	0.025
	Fourth quartile	54	46	1.837 (1.013∼3.333)	0.045
P^**^		0.035		**—**	**—**
P trend		**—**	**—**	0.014	**—**
RASSF1A is classified by median	≤Median	88	111	1	**—**
	>Median	112	89	1.551 (1.023∼2.353)	0.039
P^**^		0.021		**—**	**—**

ps: ^*^ is used unconditional Logistic regression to calculate OR and P values, Adjusted by gender, age, smoking status; ^**^ is the result of *x*^*2*^.

### Analysis of the association between telomere relative length and lung cancer

The lung cancer patient group and the control group were divided into four layers according to the quartile of telomere relative length. With the risk analysis of lung cancer with the long telomere group as the reference group, the results showed that the shortening of telomere relative length correlated with increasing risk of lung cancer(*P*_*trend*_<0.001); Then according the median divided layers, the risk of lung cancer in patients with short telomere length is 3.258 times of the long telomere length group, the difference was statistically significant as seen in Table 4.

**Table 4:** Telomere length and the risk of lung cancer

Telomere length		Lung cancer group	Control group	*OR*_*adj*_(95%*CI*)^*^	P^*^
Classified by quartile	RTL>1.27	23	80	1	—
	0.95<RTL≤1.27	47	48	2.625 (1.378∼5.002)	0.003
	0.73<RTL≤0.95	66	33	6.064 (3.164∼11.622)	<0.001
	RTL≤0.73	64	39	4.962 (2.619∼9.401)	<0.001
*P*		<0.001^**^	**—**	<0.001^***^	**—**
Classified by median	RTL>0.95	70	128	1	**—**
	RTL≤0.95	130	72	3.258 (2.118∼5.011)	0.009
*P*		<0.001^**^	**—**	**—**	**—**

ps: ^*^ is used unconditional Logistic regression to calculate OR and P values, Adjusted by gender, age, smoking status: ^**^ is the result of *x*^*2*^;^***^: is the result of trend test.

### Evaluation of lung cancer discriminative model based on 5 genetic polymorphisms

**Figure 1:**
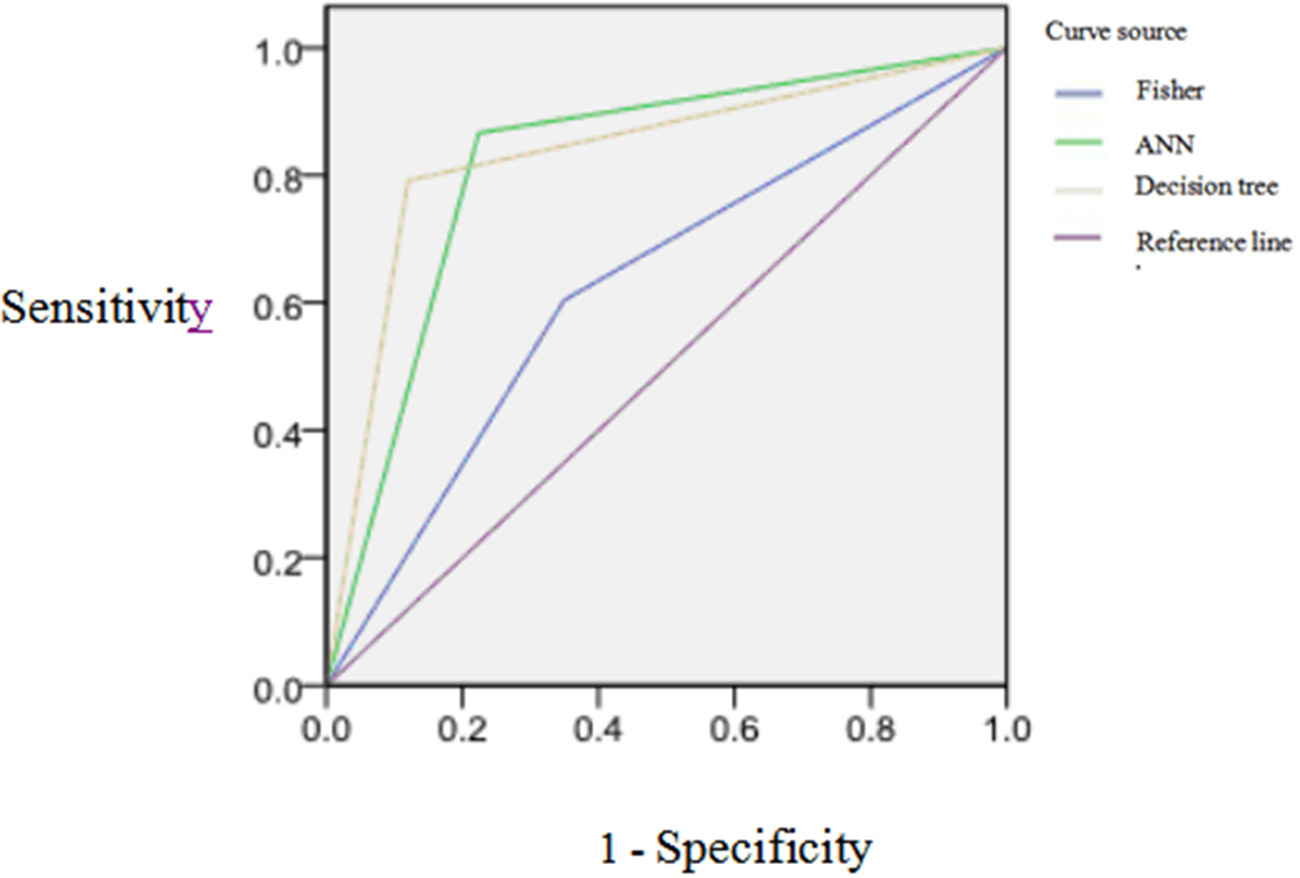
The ROC curves of three kinds of models for classification effect of prediction set model

Through analyzing the diagnostic value of three kinds of models by ROC, results showed the ROC curve area (AUC) of Fisher discriminant analysis is less than 0.7 showing the lower accurate diagnosis, but the AUC of decision tree and ANN are all closed to 0.9, showing the better accurate of diagnosis. The model prediction results are shown in Table 5 and Figure 1.

**Table 5:** The diagnostic results of the 3 models on the prediction set

Model	Sensitivity(%)	Specificity(%)	Accuracy(%)	Positive Predictive value(%)	Negative Predictive value(%)	AUC(95%CI)
Fisher	69.64	57.38	63.25	60.00	67.31	0.627 (0.570-0.684)
Decision tree	75.47	88.71	82.61	85.11	80.88	0.836 (0.792-0.879)
ANN	75.41	1	80.77	82.14	79.73	0.821 (0.776-0.866)

### Evaluation of lung cancer discriminant model based on the methylation of p16 gene and RASSF1A gene and the relative length of telomere

**Figure 2:**
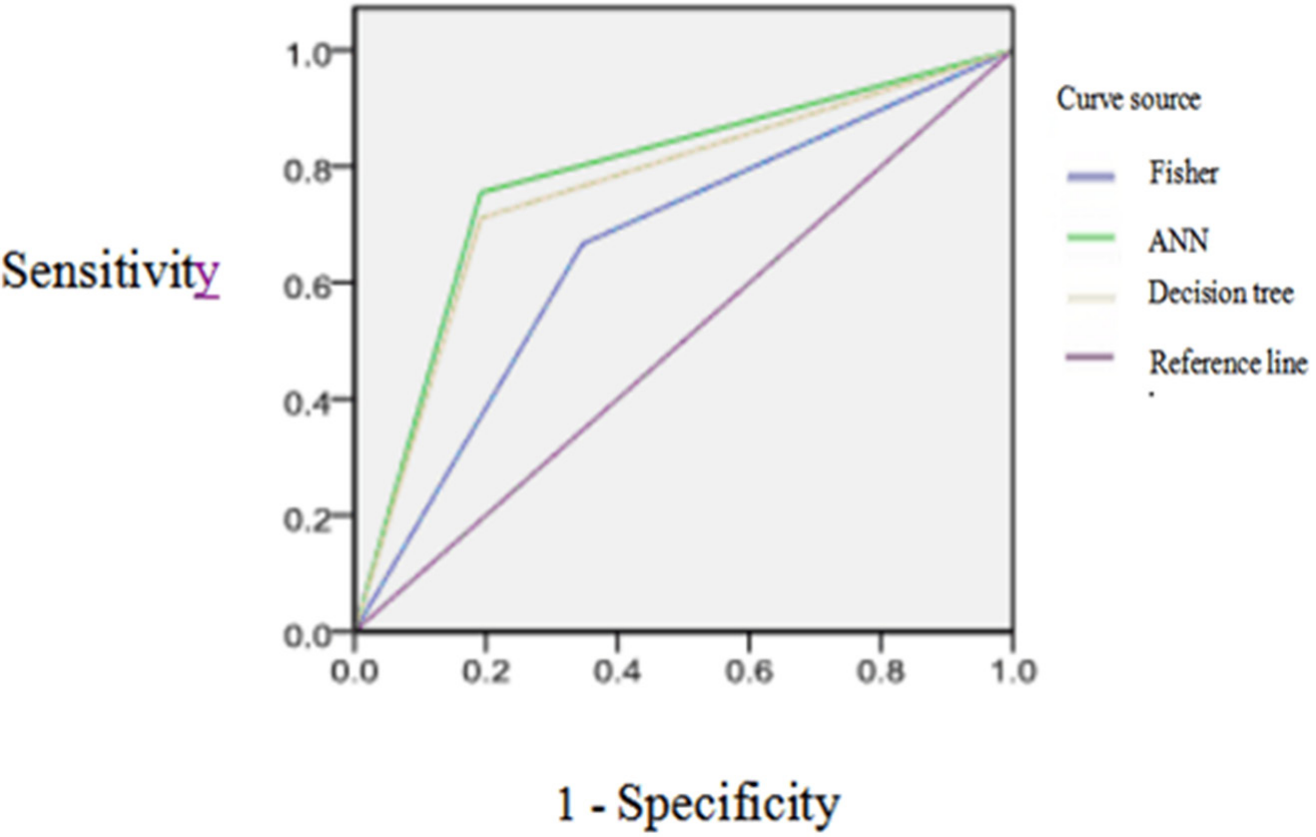
The ROC curves of three kinds of models for classification effect of prediction set model

Through analyzing the diagnostic value of models by ROC, the result showed the ROC curve area (AUC) of Fisher discriminant analysis is less than 0.7 showing the lower accurate diagnosis, but the AUC of decision tree and ANN model are more than 0.7 which indicates the moderate accurate diagnosis better than the diagnostic value of Fisher discriminant analysis as seen in Table 6 and Figure 2.

**Table 6:** The diagnostic results of the 3 models on the prediction set

Model	Sensitivity(%)	Specificity(%)	Accuracy(%)	Positive Predictive value(%)	Negative Predictive value(%)	AUC (95%CI)
Fisher	62.79	67.44	65.82	71.05	60.98	0.660 (0.551-0.770)
Decision tree	70.59	79.66	75.45	75.00	75.81	0.782 (0.686-0.878)
ANN	74.48	69.93	72.15	70.13	74.30	0.759 (0.660-0.859)

### Evaluation of lung cancer discriminant model based on tumor markers

Through random extracted 75% and 25% of samples as the training set and the prediction set, the classification accuracy rate was 72.15% and 70.59% by Fisher discriminant analysis model after repeated training. However the classification accuracy rate was 92.96% and 89.62% of decision tree C5.0 model, ANN model for prediction set and the training set classification accuracy was 92.96% and 89.62%.

**Figure 3:**
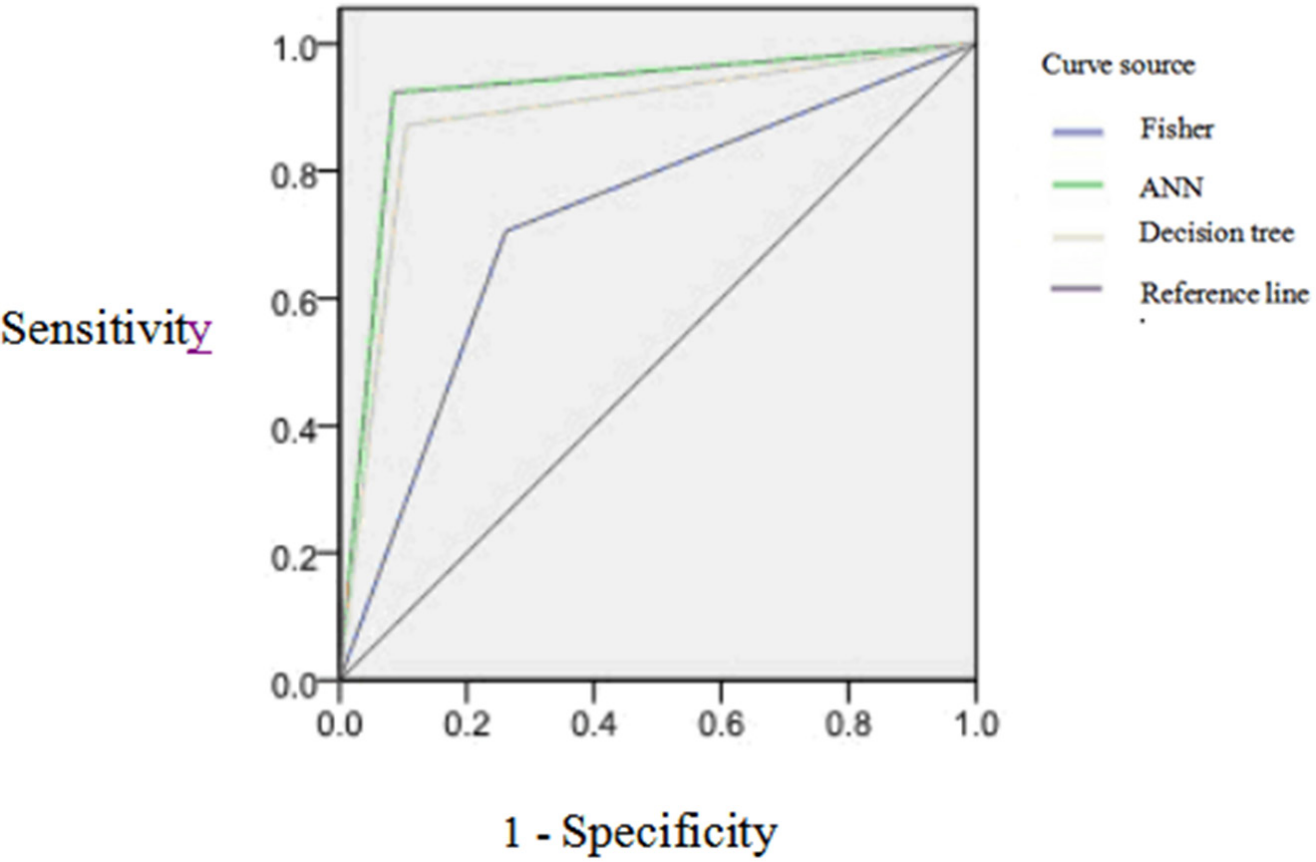
The ROC curves of three kinds of models for classification effect of prediction set model

The result of diagnostic value of models by ROC showed the AUC of Fisher discriminant analysis is 0.722, showing the moderate accurate diagnosis, the AUC of decision tree is more than 0.9, showing the better accurate diagnosis. The AUC of ANN is more than 0.9, also showing the better accurate diagnosis. Therefore, two kinds of data mining models are better than discriminant analysis model of diagnostic value. As seen in Table 7 and Figure 3.

**Table 7:** The diagnostic results of the 3 models on the prediction set

Model	Sensitivity(%)	Specificity(%)	Accuracy(%)	Positive Predictive value(%)	Negative Predictive value(%)	AUC(95%CI)
Fisher	65.38	76.00	70.59	73.91	67.86	0.722 (0.664-0.780)
Decision tree	90.70	94.74	93.00	92.86	93.10	0.929 (0.894-0.964)
ANN	89.09	90.20	89.62	90.74	88.46	0.894 (0.852-0.935)

### Classification of lung cancer in early stage (I+II stage) by using decision tree and ANN model

**Figure 4:**
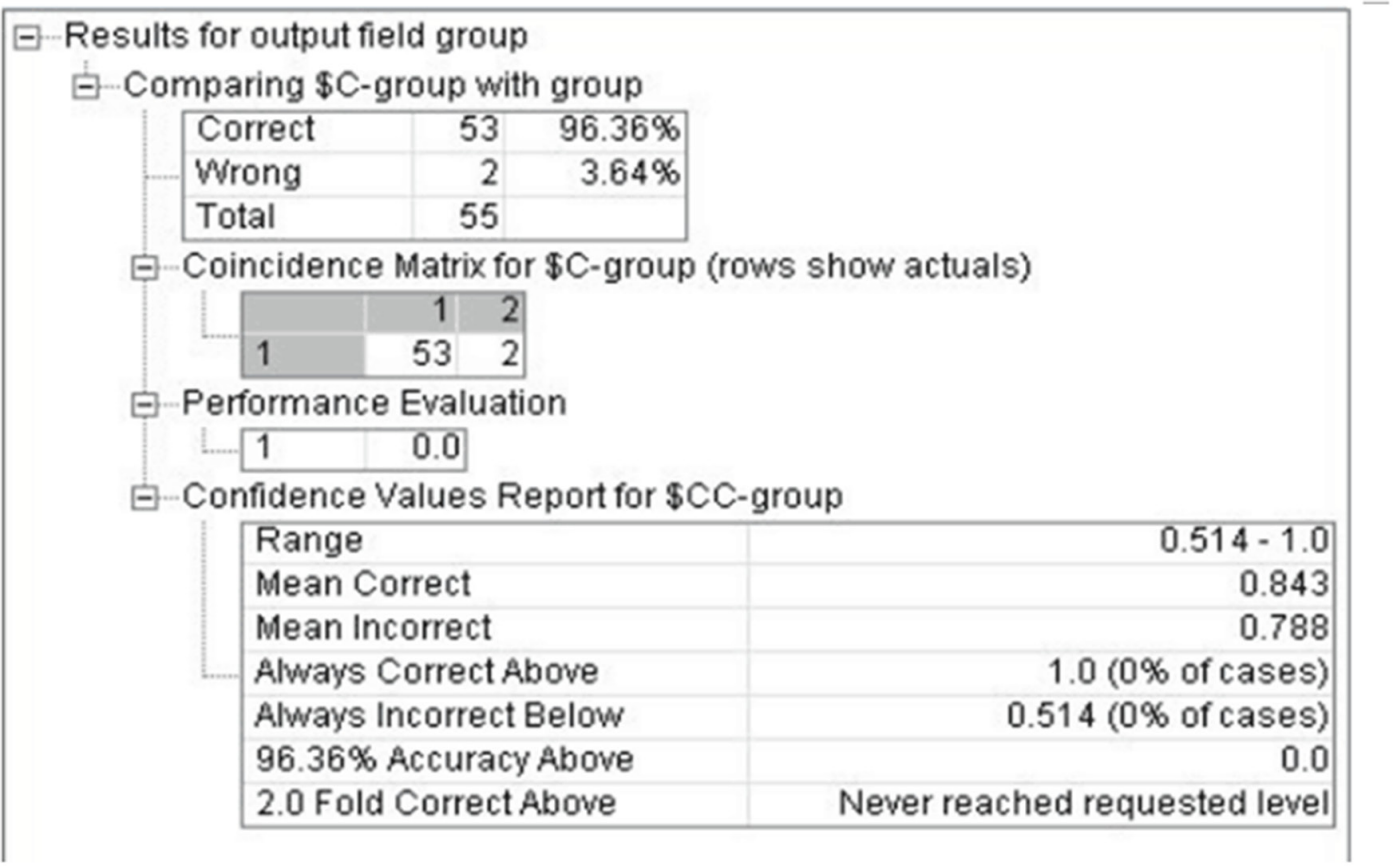
The classification of decision tree model for early stage lung cancer

**Figure 5:**
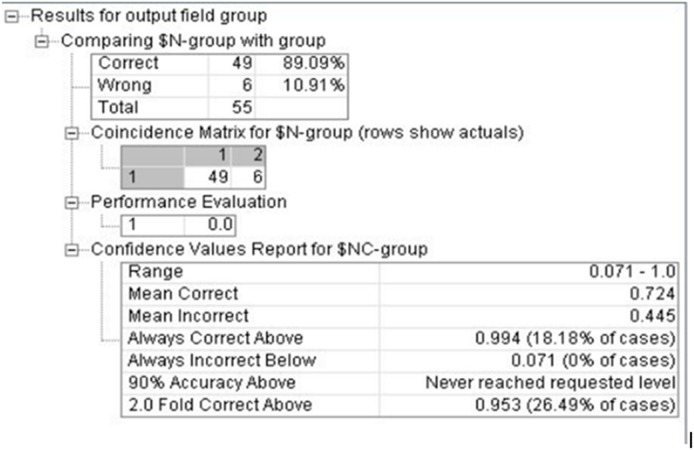
The classification of ANN model for early stage lung cancer

Through combining the genetic polymorphism of CYP1A1-rs1048943, GSTM1, mEH-rs1051740, XRCC1-rs1799782 and XRCC1-rs25489, the methylation of p16 and RASSF1A gene, the length of telomere, smoking status and other factors, the early stage classification model of lung cancer was established by using decision tree and ANN techniques through repeated training. And then we classified the lung cancer in the early stage (I+II stage), evaluated the effectiveness and diagnostic value of the model. The results shown that the classification accuracy of the decision tree model is 96.36%, the ANN model is 89.09%, which illustrated the classification results was better as seen in Figure 4 and 5.

## DISCUSSION

Recent studies have indicated that the occurrence of lung cancer is a multiple- factors and multiple-step process, and it is the result of interaction between genetic and environmental exposure factors [17]. Tumor markers can be produced directly by the tumor or by non-tumor cells. Biomarker can be found in blood, urine, or body tissues. The levels of biomarkers can be elevated specifically caused by the presence of one or more types of cancers. There are many different tumor markers, indicative of a particular disease process. They have been exploited in detection of cancers. An elevated level of a tumor marker indicates the formation or existence of cancer [18]. Therefore, such tumor makers are likely useful tools for early diagnosis, treatment and prognosis of tumor.

Therefore, genetic polymorphisms of CYP1A1-rs1048943, GSTM1, GSTT1, mEH-rs1051740 and XRCC1(rs1799782, rs25489), methylation of p16 and RASSF1A gene, and telomere length were analyzed in peripheral blood both from lung cancer patients and health controls to explore their correlation. The results showed that all indexes had different degrees of correlation with lung cancer. Smoking has the most closer relationship to lung cancer, which is consistent with other research results [19–25]. Compared with the diagnostic model based on different tumour markers, it has been shown that the AUC level of each discriminative model has been improved by about 10% based on multiple tumor markers, which indicates that the sensitivity and specificity of diagnosis can be substantially improved through combining different tumor makers compared to individual tumor marker. Therefore, multiple tumor marker analysis system is more suitable for the construction of the early intelligent discriminative model of lung cancer.

Data Mining, also called Knowledge Discovery from Database, is a complex process which extracts and excavates unknown and valuable knowledge such as model or regular pattern from mass incomplete, fuzzy, noisy, random of data [26–28]. The latest technology, such as database technology, machine learning, artificial intelligence, statistics, information retrieval and data visualization was combined together [29]. Fisher discriminant analysis is a traditional statistical classification method, the principle of this method is substitution the indicators of the observation unit based on discriminant function, obtain the corresponding discriminant function value, and finally according to the function value of the observation unit to determine the classification effect [30].

The sensitivity, specificity and accuracy of lung cancer discrimination model, based on data mining technology, were higher than Fisher discriminant analysis model, the AUC of ANN model and decision tree model are 0.929 and 0.894 respectively, which based on multiple tumour markers, but the AUC of Fisher discriminant analysis model is 0.722, which indicated that the data mining technology is more suitable for lung cancer discriminant model. Due to lack significant correlation between indexes, various factors have complicated nonlinear relationship with lung cancer. The model of Fisher discriminant analysis is a linear model, which has a higher requirement for the data, and has great limitations in analysis the variation law of the nonlinear data system [31]. The data mining technology has better intelligent characteristics when dealing with complex nonlinear data for imprecise mathematical models, and identifies and taps the relationship and potential information of indicators by automatically learning, and describe the fuzzy evaluation, therefore, the limit of data types is smaller [32, 33]. On the other hand, compared the methodology, the classification of data information by Fisher discriminant analysis, which based on the statistics attribute of samples, but the data mining technology is based on logic, which belongs to the category of intelligent machine learning.

Through further comparing two discriminant models, the sensitivity, specificity and accuracy of the decision tree model were 90.7%, 94.74%, 93%, and each index from decision tree model was better than the ANN model. The reasons probably are: firstly ANN is a processing network to deal with complex information, which composed by wide connection of many simple processing units [34], needs to transform the discrete attributes of numerical value into numerical attributes, so ANN is more susceptible to data attributes than decision trees [35]. Secondly, the neural network has the better ability to manipulate data with time sequence [36–38], but it requires more data. Moreover, it needs to draw support from the rich experience in training, the training sample set should contain all the patterns, and the input data should as far as possible haven’t relevant between each other, these lead the higher requirements of the sample data, which means the neural network is more suitable for the larger database [39]. In addition, the classification result of Decision tree model was simple, clear, intuitive structure [40–42], has more advantages in explaining and analyzing the results than ANN model.

Finally, in this study tumor markers from 55 patients with diagnosed clinical early stage (I+II) lung cancers were used to evaluate the effectiveness and diagnostic value of the model. The accuracy rate of decision tree and ANN model is 96.36 and 89.09, respectively. The diagnostic efficiency with the new model was better than ANN model.

Limitations need to be considered in explaining the time and causal relationship between the occurrence of molecular events and lung cancer, although we tried to recruit more cases who on Clinical stage I and II, the inherently limitations of case-control design still exist. In the next step, with the permission of funds and technology, we will verify the efficiency of the diagnosis model by expanding the sample size and/or using prospective studies.

The early intelligent discriminative models of lung cancer has the better diagnostic effect and profound significance for diagnostic the early stage lung cancer, which based on multiple tumour markers and data mining techniques.

## MATERIALS AND METHODS

### Subject of study

The patients (n=200) were diagnosed as lung cancer by pathology from the First Affiliated Hospital of Zhengzhou University, including 87 squamous cell carcinoma cases, 72 adenocarcinoma cases, 33 small cell lung cancer cases and 8 large cell lung cancer cases; 55 cases of Clinical stage I and II, 145 of clinical stage III and IV; Age: 59.56 ± 10.56 years old; Gender: 143 Male and 57 female; Smoker: 107 smokers and 93 non-smokers; The control group (n=200) was from healthy non-tumor persons by Physical examination department from the First Affiliated Hospital of Zhengzhou University: Age (53.70±13.34) years old; 151 Male cases, 49 female cases, 79 smokers cases, 121 non-smokers; 2ml of peripheral blood was collected after morning fasting. Epidemiological data and blood samples were collected by professional investigators and doctors after the subject’s informed consent.

### Each index detection method

Genomic DNA was extracted from 2 ml blood according to the instruction of the QIAamp DNA Mini kit.

The polymorphisms were detected with allele specific amplification method (ASA) and polymerase chain reaction-restriction fragment length polymorphism (PCR-RFLP) method. The genes included CYP1A1 rs1048943ll, rs35463883 [43], mEH rs1051740, rs1051740 [44], XRCC1 rs1799782, rs25487 [45], rs25489 [46]. The polymorphisms of GSTM1 and GSTT1 genes were detected by Multiple PCR method [47].

The methylation level of p16 and RASSF1A were detected by real-time methylation specific PCR [48–49]. The relative telomere length was detected by real-time fluorescence quantitative PCR method. GAPDH was used as a reference gene [50].

### General statistical analysis of data

The general statistical analysis was assessed by SPSS21.0 software, according statistical data type to choose description method, using mean±standard deviation when data was normal distribution, using median and inter-quartile range when data wasn’t normal distribution, comparing count data groups used Student’s t test or Wilcoxon rank sum test; Comparing count data groups used chi-square test, the correlation between indicators and lung cancer was determined using the logistic regression. *α*=0.05.

### Data mining model establishment

All the data are normalized to [0, 1] with the max min method.

According to the proportion of 3:1, the data is divided into training set and prediction set by SPSS Clementine software of random sampling founction.

Based on Clementine SPSS 12 software of fisher discriminant analysis, decision tree C5.0 and BP neural network algorithm, the diagnostic model of lung cancer was established.

The model was evaluated with diagnostic test, the indexes include sensitivity, specificity, accuracy, area under the receiver operating characteristic curve (AUC), positive predictive value and negative predictive value. The AUC less than 0.5 shows the diagnosis hasn’t significance; the AUC between 0.5∼0.7 showing the lower accurate diagnosis; AUC between 0.7∼0.9 showing the medium accurate diagnosis; AUC more than 0.9, showing the higher accurate diagnosis.

## Abbreviations

DM: Data mining
ANN: Artificial Neural Networks
KDD: Knowledge Discovery from Database
ASA: allele-specific amplification
PCR: Polymerase Chain Reaction
RFLP: Restriction fragment lebgth polymorphism
Qmsp: real-time methylation specific PCR
BP: Back-Propagation
GWAS: genome wide association study
SNP: Single nucleotide polymorphisms
BER: base excision repair
AUC: area under the curve
CYP1A1: Cytoehrome P4501A1
GST: Glutathione-S-transferase
mEH: microsomal epoxide hydrase
XRCC1: X-ray repair cross-complementing gene 1
CGI: CpG island
NSCLC: non-small cell lung cancer
TM: Tumor Marker
OR: Odds Ratio
CI: Confidenee Interva
